# The Secret Life of Giant Viruses in the California Current

**DOI:** 10.1128/mSystems.00751-21

**Published:** 2021-07-13

**Authors:** Jônatas Santos Abrahão

**Affiliations:** a Universidade Federal de Minas Gerais, Microbiology Department, Virus Laboratory, Minas Gerais, Brazil

**Keywords:** *Nucleocytoviricota*, *Varidnaviria*, giant virus, oceans, protists, transcriptomics

## Abstract

In the last few decades, the virology field has experienced a revolution in knowledge related to viral richness, diversity, and distribution in the oceans. Metagenomics associated with virus isolation methods have contributed to outstanding discoveries in marine virology. Giant viruses and other protist-infecting viruses belonging to the phylum *Nucleocytoviricota* have raised fundamental questions such as “what are the limits of virion size?”, “what is a viral genome able to encode?”, and “what is the ecological role of giant viruses in the ocean?” In a recent paper published in *mSystems* by Ha, Moniruzzaman, and Aylward (mSystems 6:e00293-21, 2021, https://doi.org/10.1128/mSystems.00293-21), the authors demonstrated by metatranscriptomic-related analyses that giant viruses are active members of the California Current microbial community, replicating, modulating, and exchanging genes with their protist hosts. This work not only explores the dynamics of giant virus gene expression in a natural environment but also reveals that nucleocytoviricotal abundance and ecological importance are underestimated.

## COMMENTARY

Due to the impact caused by viruses in human history, virology as a science was mostly developed due to anthropocentric motivations. This fact can partially explain why, during the past decades, Homo sapiens and organisms related to human interests (e.g., agricultural plants and farm animals) were the species with which most known viral species were associated (1). Yet, in theory, every cellular organism species is infected and influenced by viruses (2). Viruses are widespread and represent the most abundant biological entities on Earth (with approximately 10^31^ viruses) (2, 3). In recent decades, an increasing amount of data has pointed to viruses as fundamental players acting in biological networks and nutrient cycling in oceans (4). Seminal works on marine virology described the presence of viruses of bacteria and, subsequently, the first known protist-infecting viruses (5–7). Just two decades ago, the first metagenomic studies in oceans revealed an overwhelming abundance and richness of viruses in coastal waters (8). Since then, ocean metagenomics has become a powerful tool for large- and local-scale studies of environmental virology. Therefore, in recent years, studies on marine virology have intensified, exploring virological information both by metagenomics and by the improvement of systems for the isolation of new viruses.

In this effervescent context, the serendipitous discovery of giant viruses capable of infecting protists inaugurated a new chapter in the history of virology. In 2003, the description of an amoebal-infecting virus presenting a gigantic and complex particle (800 nm) contradicted the definition of viruses as “filtrable agents.” The so-called mimivirus was just the first to be discovered of the collection of spectacular amoebal-infecting viruses that have been described in recent years (9). Related viruses have already been described infecting other protists, further expanding the knowledge regarding the richness of giant viruses (10). The size of giant virus capsids is impressive and can reach more than 2 μm (>100 virion proteins), as observed in tailed tupanviruses and oval-shaped pithoviruses (11, 12). Giant virus genomes present an even more unforeseen complexity. To date, the Pandoravirus genome can reach 2.7 megabases and approximately 90% ORFan genes (i.e., genes with no homologs in databases) (13). The description of categories of genes never seen before in the virosphere represents the cherry on the top of giant virus genomics, including translation-related genes (aminoacyl tRNA synthetases, tRNAs, initiation/elongation/termination factors) and genes related to glycosylation, photosynthesis, fermentation, and the Krebs cycle (11, 14, 15). Metagenomic studies have demonstrated the ubiquity of giant viruses in different environments and have been a source of comprehensive works on giant virus richness and diversity, mostly clustered as members of the phylum *Nucleocytoviricota* (2, 15). As the readership will notice, this new chapter in virology history has begun to be written very recently and, despite all the aforementioned breathtaking discoveries, a plethora of new and relevant questions have been raised.

In a recent paper published in *mSystems* (16), Ha, Moniruzzaman, and Aylward addressed one of those fundamental questions by investigating the magnitude of actively replicating *Nucleocytoviricota* viruses in a marine habitat. Using an innovative and elegant 2.5-day metatranscriptomic time-series experimental design, the authors investigated gene expression related to those viruses at surface waters in the California Current using a database with 2,436 annotated *Nucleocytoviricota* genomes. Remarkably, a high level of gene expression was detected for 145 *Nucleocytoviricota* genomes, mostly belonging to the orders *Imitervirales* and *Algavirales*, which are viruses that infect a wide range of algae and other protists. Such data reinforce the notion that a substantial part of protist cells present in the oceans are truly virocells, infected and subverted by *Nucleocytoviricota* viruses (17). Interestingly, the authors present data suggesting that viral activity may be higher at night for some of those viruses, raising questions about the diel influence on host lifestyle (growth and division) and viral gene expression.

In addition to the expected high expression of viral core genes, the work revealed that marine giant viruses mitigate cellular stress by expressing superoxide dismutase homologs. The detection of transcripts related to central metabolic enzymes (i.e., genes involved in glycolysis, the TCA cycle, the pentose phosphate pathway, and beta oxidation) highlights that *Nucleocytoviricota* viruses are able to reprogram cell metabolism to optimize their fitness. Translation-related genes represent a trademark of some *Mimiviridae* family members, and the detection of their transcripts reinforces that these genes are truly expressed by *Nucleocytoviricota* viruses in natural environments, and may represent one more resource to improve their fitness. In addition, the authors present data confirming the expression of several transcripts related to cytoskeletal proteins, including actin, myosin, and kinesin. Remarkably, previous studies have identified cytoskeletal-related homologs in *Nucleocytoviricota* genomes (18–20). Ha and colleagues contributed to this knowledge by presenting 109 myosin and 200 kinesin homologs. Those results may suggest that such genes are widespread in *Nucleocytoviricota* genomes and may be used by those viruses to control cell shape and viral factory development.

At the occasion of the mimivirus discovery, one could have thought that giant viruses were rare and an exception in the virosphere. However, today, just 18 years later, it is crystal clear that giant viruses are underestimated components of viral richness and diversity in the oceans. The work of Ha, Moniruzzaman, and Aylward is inspiring not only for reinforcing this point but also for demonstrating by metatranscriptomics that viruses of protists are actively and abundantly replicating, modulating, and exchanging genes with their hosts in the California Current biome. This exciting journey through the ocean’s secrets reveals more about the lifestyle of this fabulous group of viruses.
